# Systemic Mastocytosis with Associated Chronic Lymphocytic Leukemia: A Matter of Diseases or Prognostic Factors?

**DOI:** 10.4274/tjh.2017.0014

**Published:** 2017-08-02

**Authors:** Antonella Zagaria, Luisa Anelli, Nicoletta Coccaro, Giuseppina Tota, Claudia Brunetti, Angela Minervini, Paola Casieri, Luciana Impera, Crescenzio Francesco Minervini, Annamaria Giordano, Paola Orsini, Cosimo Cumbo, Giorgina Specchia, Francesco Albano

**Affiliations:** 1 Bari University Faculty of Medicine, Department of Emergency and Organ Transplantation, Bari, Italy

**Keywords:** Systemic mastocytosis, Systemic mastocytosis with an associated hematological neoplasm, Chronic lymphocytic leukemia, KIT D816V

## To The Editor,

Systemic mastocytosis (SM) is a clonal disorder characterized by the accumulation of abnormal mast cells in various tissues and by a broad range of diseases, ranging from indolent to advanced systemic pathology. In 2016, the World Health Organization revised the SM classification into seven distinct variants [1]; among them is SM with an associated hematological neoplasm (SM-AHN), accounting for approximately 40% of all SM cases [2]. Usually the AHN is an aggressive neoplasm that can be challenging to diagnose either because the mast cell infiltrate can be subtle and difficult to identify or because the mast cell infiltrate can be prominent, thus obscuring the underlying AHN. Frequently the AHN is represented by a myeloid neoplasm, whereas the observation of an association between SM and lymphoproliferative diseases is uncommon. In particular, chronic lymphocytic leukemia (CLL) in the context of SM-AHN has been rarely reported [2,3,4,5,6] (Table 1). Here, we describe a new case of SM with associated CLL, in which the cytological features of the two neoplasias were evident in bone marrow aspirate; moreover, in this case molecular analysis specific for SM was performed.

**Figure 1:**
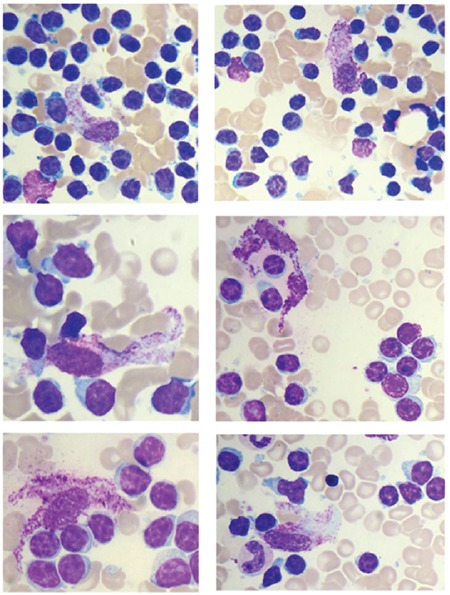
Systemic mastocytosis with an associated hematological neoplasm bone marrow aspirate. Atypical mastocytes with spindle nucleus and granules spread throughout the plentiful basophilic cytoplasm. The mastocytes are located in the context of bone marrow rich in mature lymphocytes.

A 65-year-old female patient presented with lymphocytosis (leukocytes, 66x10^9^ cells/L; lymphocyte absolute count, 63x10^9^ cells/L; hemoglobin level, 12.1 g/dL; platelets, 243x10^9^ cells/L). Physical examination revealed no abnormalities. Peripheral blood smear analysis demonstrated the presence of ~90% small mature lymphocytes together with smear cells (Gumprecht shadows). Flow cytometry analysis of the lymphocyte population showed positive staining for anti-CD5, CD19, CD23, and CD20 antibodies and a k-type light chain restriction. The bone marrow aspirate appeared hypercellular due to the infiltration of lymphoid cells, which were morphologically similar to those in the peripheral blood and accounted for at least 70% of all nucleated marrow cells. Moreover, morphologic analysis showed the presence of at least 10% of mast cells, mainly characterized by a fusiform cytoplasm (Figure 1). Bone marrow biopsy confirmed the lymphocyte infiltration associated with the presence of atypical mast cells (CD117^+^, tryptase+, CD2^+^, CD25^+^); multifocal mast cell aggregates (>15 mast cells) were frequently observed in perivascular and paratrabecular bone marrow locations. The tryptase level was 64.5 ng/mL. Total body computed tomography revealed no abnormalities. Conventional cytogenetics showed a normal karyotype, i.e. 46,XX [20], whereas fluorescent in situ hybridization, performed with probes specific for chromosomal aberrations associated with CLL, revealed the presence of del(13)(q14) and no alterations at the 17p13, 11q22, and 12q13 loci. IGVH was not mutated. Molecular analysis revealed the KIT D816V mutation, whereas SRSF2, ASXL1, and JAK2 V617F were not altered. Based upon these findings, the patient was diagnosed with SM (the major criterion and 3 minor criteria) with associated CLL (Rai 0, Binet A stage). Since neither of the two diseases showed signs of activity it was decided to choose a “watch and wait” strategy, and 1 year after the diagnosis the SM-AHN remains stable (lymphocyte absolute count, 92x10^9^ cells/L; tryptase level, 67 ng/mL) and the patient is doing well. Reportedly, SM-AHN has a poor prognosis with a median survival of less than 2-4 years [7]. Given the low frequency of CLL in the context of SM-AHN, it is very difficult to draw general prognostic considerations for this entity, but recently it was published that advanced SM patients bearing SRSF2 and/or ASXL1 mutations have a poor prognosis [8,9]. Note that among the SM patients reported in those two studies there was not a case associated with CLL. In conclusion, we suggest that the stable course of SM-AHN may derive from the concurrence of the positive prognostic factors that characterize the two diseases in our patient.

## Figures and Tables

**Table 1:**
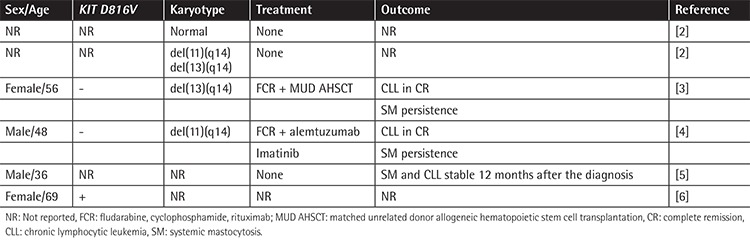
Main characteristics of prior reported SM-CLL cases.
